# Nanosize Control on Porous β-MnO_2_ and Their Catalytic Activity in CO Oxidation and N_2_O Decomposition

**DOI:** 10.3390/ma7053547

**Published:** 2014-05-06

**Authors:** Yu Ren, Zhen Ma, Sheng Dai

**Affiliations:** 1 National Institute of Clean-and-low-carbon Energy, Beijing 102211, China; 2 School of Chemistry and EaStChem, University of St Andrews, St Andrews, Fife KY 16 9ST, UK; 3 Shanghai Key Laboratory of Atmospheric Particle Pollution and Prevention (LAP^3^), Department of Environmental Science and Engineering, Fudan University, Shanghai 200433, China; 4 Chemical Sciences Division, Oak Ridge National Laboratory, Oak Ridge, TN 37831, USA; E-Mail: dais@ornl.gov

**Keywords:** mesoporous, pyrolusite, CO oxidation, N_2_O decomposition, textural properties

## Abstract

A major challenge in the synthesis of porous metal oxides is the control of pore size and/or wall thickness that may affect the performance of these materials. Herein, nanoporous β-MnO_2_ samples were prepared using different hard templates, e.g., ordered mesoporous silica SBA-15 and KIT-6, disordered mesoporous silica, and colloidal silica. These samples were characterized by Powder X-Ray Diffraction (PXRD), Transmission Electron Microscopy (TEM), and N_2_ adsorption-desorption. The pore size distribution of β-MnO_2_ was tuned by the different hard templates and their preparation details. Catalytic activities in CO oxidation and N_2_O decomposition were tested and the mesoporous β-MnO_2_ samples demonstrated superior catalytic activities compared with their bulk counterpart.

## 1. Introduction

The 21st century has already presented human society with many challenges: greenhouse gas emission control, energy conservation, cleaner chemical processing, *etc*. Mesoporous transition metal oxides possess d-shell electrons confined to nanosized walls, redox active internal surfaces, and ordered pore networks, thus generating a great deal of interest for catalysis [1–3], separation or storage of ions/molecules [4–8], and energy conversion and storage [9,10]. While it is possible to synthesize mesoporous transition metal oxides exhibiting highly ordered pore structures with a variety of symmetries, tailoring the pore size/wall thickness to particular values is challenging [11]. However, being able to do so is key to the functionality of porous solids [12–15].

The use of hard templates, e.g., mesoporous silicas (from which transition metal oxide replicas may be cast), has delivered the combination of highly ordered pore stuctures with crystalline walls. Since the walls of the template define the pores of the mesoporous transition metal oxide, it is necessary to control the thickness of the template walls in order to prepare a variety of pore sizes for the target material. The pore size and wall thickness of mesoporous silicas change with the hydrothermal synthesis conditions and this has been used to prepare mesoporous silicas with different pore sizes [16–19]. The pore size and wall thickness of mesoporous silicas can also be tuned by varying the calcination temperature of these materials [20,21], and the pore sizes and wall thicknesses of casted mesoporous transition metal oxides can also be changed by using these mesoporous silicas as hard templates [22]. Due to the limitation of controlled size range, other templates, such as colloidal silica or alkaline ions, have also been employed to prepare mesoporous metal oxides with pore size extending to 30 nm or above [14,23]. Here we present results demonstrating the textural properties control over mesoporous manganese oxides, β-MnO_2_, which can be prepared with pore sizes in the range from 3.3 to 28 nm and wall thicknesses ranging from 5 to 30 nm. The catalytic performance of these catalysts was studied using CO oxidation and N_2_O decompostion as probe reactions.

## 2. Results and Discussion

### 2.1. Textural Properties’ Control over Mesoporous β-MnO_2_

Recently, 3D mesoporous β-MnO_2_ samples have been prepared with pore sizes ranging from 3.4 to 28 nm in diameter, with wall thicknesses from 4.7 to 30 nm; the influence of size on the rate of Li-intercalation (as the cathode for Li-ion batteries) has therefore been studied [14]. Here we re-prepared and re-characterized some mesoporous β-MnO_2_ samples and studied the possible correlation between their textural properties and the catalytic reaction (CO oxidation and N_2_O decomposition). In the current contribution, there are three categories of mesoporous β-MnO_2_: 3D ordered mesoporous β-MnO_2_ templated by KIT-6 silica (β-MnO_2_-*X*, *X* stands for the hydrothermal treatment temperature of KIT-6 templates, see Experimental Section); 1D ordered mesoporous β-MnO_2_ templated by SBA-15 silica (β-MnO_2_-1D-100); and 3D disordered mesoporous β-MnO_2_ (β-MnO_2_-d4 and β-MnO_2_-d30, here d stands for “disordered”) templated by disordered mesoporous silica and colloidal silica. The detailed preparation procedures are described in Section 3.

**Figure 1.**
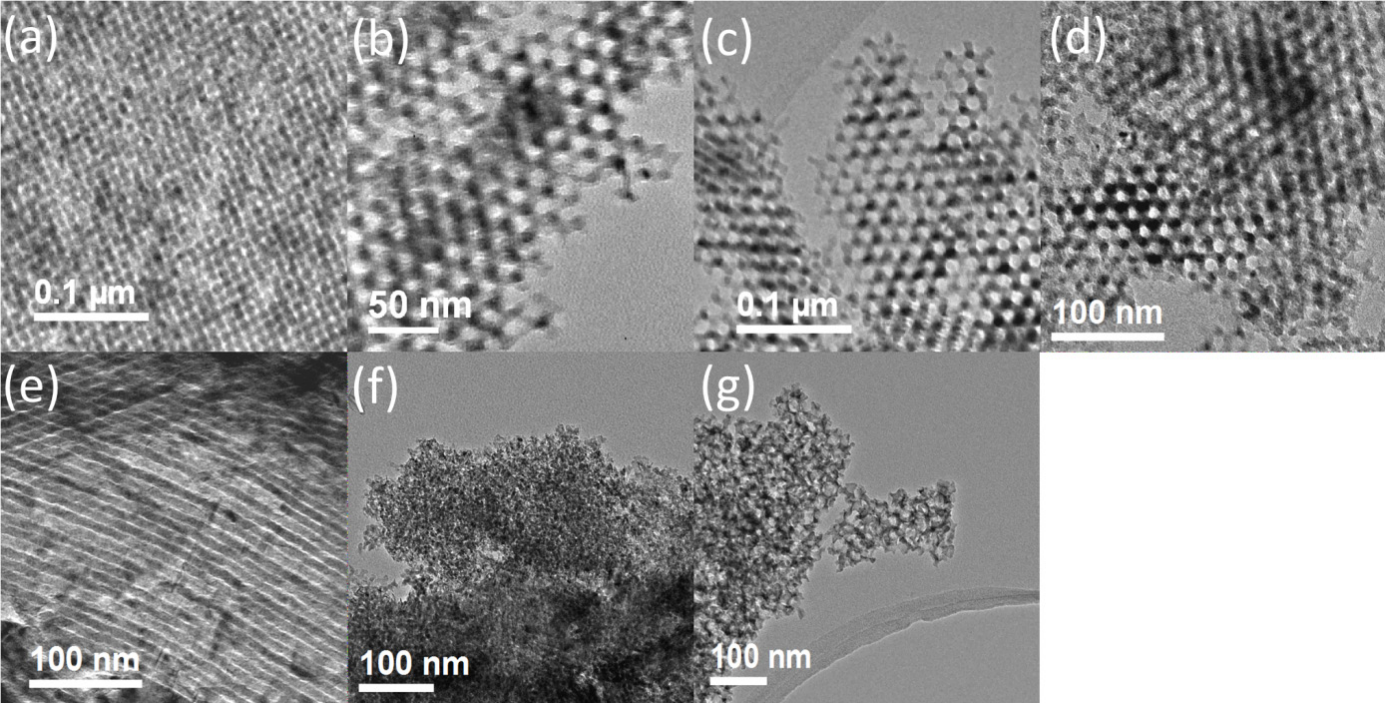
TEM images of different crystalline mesoporous β-MnO_2_ materials prepared using different hard templates: (**a**) β-MnO_2_-60; (**b**) β-MnO_2_-80; (**c**) β-MnO_2_-100; (**d**) β-MnO_2_-100B; (**e**) β-MnO_2_-1D-100; (**f**) β-MnO_2_-d4; and (**g**) β-MnO_2_-d30.

TEM data for the mesoporous β-MnO_2_ are presented in Figure 1. In each case, many regions of the sample were examined and the TEM images presented are representative of the materials as a whole. 3D ordered mesoporous β-MnO_2_ of β-MnO_2_-60, β-MnO_2_-80, β-MnO_2_-100, and β-MnO_2_-100B have ordered mesostrucutre with Ia3d symmetry (Figure 1a–d), while β-MnO_2_-1D-100 demonstrates the P6mm structure (Figure 1e). The other two mesoporous β-MnO_2_, β-MnO_2_-d4 and β-MnO_2_-d30, have disordered mesostructures (Figure 1f,g): the former has a worm-like mesostructure, while the latter has the hole mesostructure, resulting from the colloidal silica (Ludox AS-40) template.

**Figure 2.**
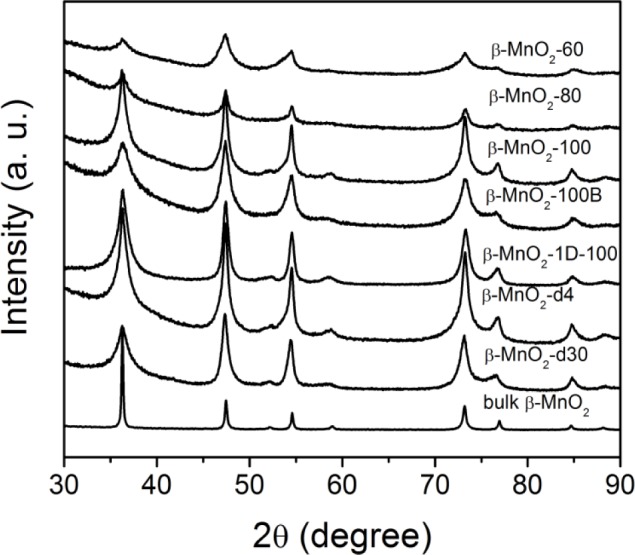
Wide-angle PXRD patterns of different crystalline mesoporous β-MnO_2_.

The wide angle PXRD data for different mesoporous β-MnO_2_ are shown in Figure 2. The data confirm the β-MnO_2_ phase (pyrusite, ICDD 00-024-0735), in good agreement with the bulk one (Aldich). The breadth of the diffraction peaks from mesostructured β-MnO_2_ is due to the nanosized pore wall.

**Figure 3.**
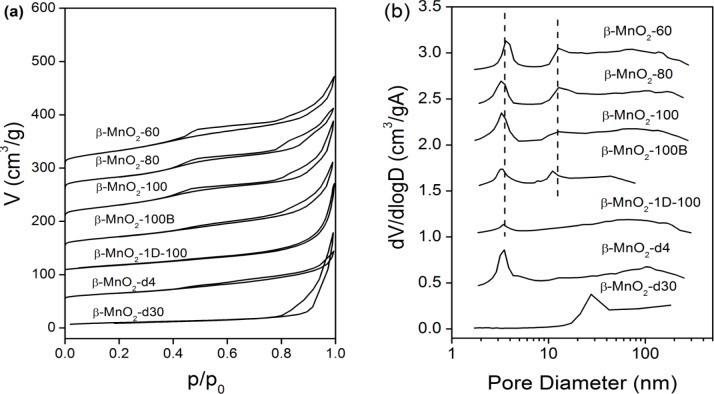
N_2_ adsorption-desorption isotherms (**a**) and pore size distributions (**b**) for the crystalline mesoporous β-MnO_2_ materials using different silica as the template. The isotherms for β-MnO_2_-60, β-MnO_2_-80, β-MnO_2_-100, β-MnO_2_-100, β-MnO_2_-1D-100, β-MnO_2_-1D-d4, and β-MnO_2_-d30 are offset vertically by 300, 250, 180, 150, 100, 50, and 0 cm^3^/g, respectively. The pore size distribution for β-MnO_2_-60, β-MnO_2_-80, β-MnO_2_-100, β-MnO_2_-100, β-MnO_2_-1D-100, β-MnO_2_-1D-100, β-MnO_2_-d4, and β-MnO_2_-d30 are offset vertically by 2.8, 2.4, 2.0, 1.5, 1.0, 0.5, and 0 cm^3^/g·Å, respectively.

The N_2_-sorption data for mesoporous β-MnO_2_ are shown in Figure 3. The adsorption-desorption isotherms for each mesoporous β-MnO_2_ are shown in Figure 3a. They correspond to a type IV isotherm with a H1 hysteresis loop [24]. Pore sizes, wall thicknesses, pore volumes, and surface areas are presented in Table 1. The BET surface area of this series of mesoporous β-MnO_2_ is relatively small compared with those reported previously [14]. The wall thickness of the ordered mesoporous β-MnO_2_, β-MnO_2_-60, β-MnO_2_-80, and β-MnO_2_-100 increase when increasing the hydrothermal temperature of mesoporous SiO_2_. The pore diameters and surface area of the ordered mesoporous β-MnO_2_ virtually do not change with the corresponding hydrothermal temperature of mesoporous SiO_2_. Considering the pore size distribution, Figure 3b, mesoporous β-MnO_2_ samples templated by KIT-6 (β-MnO_2_-60, β-MnO_2_-80, β-MnO_2_-100, and β-MnO_2_-100B) exhibit two peaks, and the pore volume of the larger mesopore increases in proportion with the decrease in the hydrothermal temperature. For instance, the pore volume ratio of 12.8 nm pore to 3.4 nm pore is 0.80 and 0.75 for β-MnO_2_-60 and β-MnO_2_-80, respectively, larger than that of β-MnO_2_-100 (0.43). It is known that the larger mesopore (12–14 nm) arises when the microporous bridges linking the two sets of pores in KIT-6 are broken, resulting in the filling of one or other set of pores—but not both simultaneously [11,14,23,25,26]. We conclude that the lower hydrothermal temperarture must reduce the micro-bridges, resulting in the appearance of the larger pores in the replica β-MnO_2_ structure. Meanwhile, decreasing Mn(NO_3_)_2_/KIT-6 ratio during the impregnation can also facilitate the formation of large mesopores [14].

### 2.2. CO Oxidation and N_2_O Decomposition over Mesoporous Manganese Oxide Catalysts

**Figure 4.**
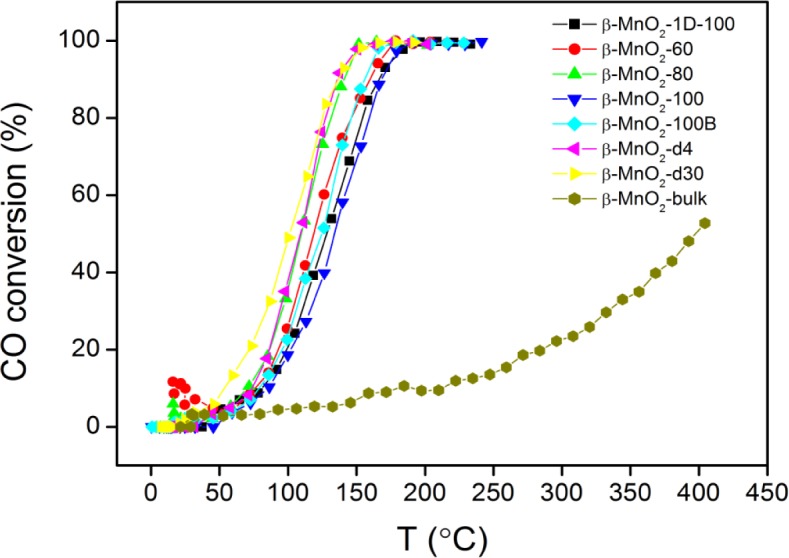
CO conversions on mesoporous β-MnO_2_ samples. Catalyst weight: 50 mg; CO concentration: 1% (in air); flow rate: 37 mL/min.

Different mesoporous β-MnO_2_ samples were tested in CO oxidation, and a commercial β-MnO_2_ (bulk β-MnO_2_) was also used for comparison. As shown in Figure 4, bulk β-MnO_2_ is not particularly active in CO oxidation, achieving 50% CO conversion at 400 °C. On the other hand, other mesoporous β-MnO_2_ samples start to show CO conversions above 50 °C and reach complete conversion at 200 °C. The T_50_ (temperature requiured for 50% conversion) values of mesoporous β-MnO_2_ samples are in the range of 102–134 °C. β-MnO_2_-d30 is the most active, whereas β-MnO_2_-100 is the least active for CO oxidation. The high activity of mesoporous β-MnO_2_ samples is ascribed to the higher surface area (30–135 m^2^/g) of these samples than that of bulk β-MnO_2_ (0.5 m^2^/g).

**Figure 5.**
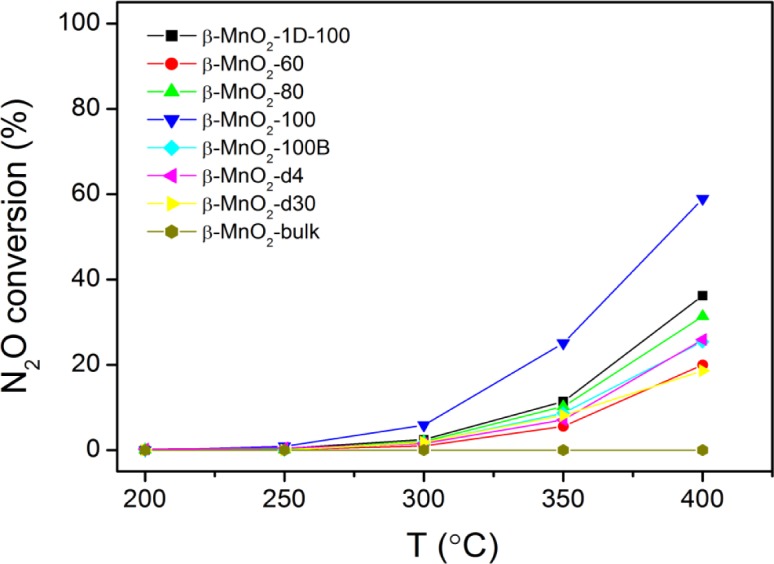
N_2_O conversions of mesoporous β-MnO_2_ samples. Catalyst weight: 0.5 g; N_2_O concentration: 0.5% (in He); flow rate: 60 mL/min.

The conversion of N_2_O over different mesoporous β-MnO_2_ is shown in Figure 5. Bulk β-MnO_2_ is not active at all even at 400 °C, whereas mesoporous β-MnO_2_ samples are active at temperatures higher than 250 °C, achieving conversions between 15% and 60% at 400 °C. The trend is consistent with the trend observed in CO oxidation, *i.e.*, mesoporous β-MnO_2_ samples are more active than bulk β-MnO_2_. However, for mesoporous β-MnO_2_ samples, the activities in N_2_O decomposition followed the sequence of β-MnO_2_-d30~β-MnO_2_-60 < β-MnO_2_-d4~β-MnO_2_-100B < β-MnO_2_-80 < β-MnO_2_-1D-100 < β-MnO_2_-100. The activity sequence is almost the reversal of the trend seen in CO oxidation. This could be because CO oxidation needs the adsorption of O_2_ on the catalyst surface, whereas N_2_O needs the desorption of O_2_ from the catalyst surface.

## 3. Experimental Section

### 3.1. Materials

Mn(NO_3_)_2_·4H_2_O (98%), bulk β-MnO_2_ (micron sized, 99.9%), concentrated HCl (37%), NaOH (99.3%), Pluronic P123 (*M*_n_ = 5800), 1-butanol (99.4%), absolute ethanol (99.9%), Ludox AS-40 colloid silica (40%), and tetraethyl orthosilicate (98%) were all purchased from Sigma-Aldrich (St Andrews, UK).

### 3.2. Preparation

The synthesis of the mesoporous silica KIT-6 was based on the procedure described previously [17,18]. The mesoporous silicas with different pore size prepared here are denoted as KIT-*X*, where *X* corresponds to the hydrothermal treatment temperature. The preparation of disordered mesoporous silica with a pore diameter of *ca.* 8 nm was based on the aforementioned procedure [14].

The synthesis of the two-dimensional mesoporous silica SAB-15 was based on the procedure described previously [16]. KIT-60, KIT-80, KIT-100, SBA-15, and the disordered ~8 nm mesoporous silica were used as the hard templates to prepare crystalline mesoporous β-MnO_2_ following the procedure described in a previous report [14]. Typically, 30 g Mn(NO_3_)_2_·6H_2_O (98%) was dissolved in 20 mL water to form a saturated solution. 5 g mesoporous silica was dispersed in 200 mL dried *n*-hexane. After stirring at room temperature for 3 h, 5 mL of the saturated Mn(NO_3_)_2_ solution was added slowly with stirring. The mixture was stirred overnight, filtered and dried at room temperature until a completely dried powder was obtained. The sample was heated slowly to 400 °C at a rate of 1 °C/min, calcined at that temperature for 3 h, and after cooling to room temperature, the resulting material was treated twice with a 2 M hot NaOH solution, followed by washing with water several times and drying at 60 °C. The obtained mesoporous β-MnO_2_ was named β-MnO_2_-60, β-MnO_2_-80, β-MnO_2_-100, β-MnO_2_-1D-100, and β-MnO_2_-d4 (disordered β-MnO_2_ with pore size of *ca*. 4 nm) using the KIT-60, KIT-80, KIT-100, SBA-15, and the disordered ~8 nm mesoporous silica as the hard template, respectively.

The preparation of ordered mesoporous β-MnO_2_ with a relatively higher proportion of large mesopore (denoted as β-MnO_2_-100B) followed a previously reported procedure [25]. 4 g Mn(NO_3_)_2_·6H_2_O (98%) was dissolved in 150 mL ethanol, followed by the addition of 5 g mesoporous silica, KIT-100. After stirring at room temperature until all the solution had been absorbed, the powder was re-dispersed in 100 mL dry *n*-hexane under stirring in an open beaker. Once all the solvent had evaporated, the sample was heated slowly to 400 °C at a rate of 1 °C/min and calcined at that temperature for 3 h. After cooling to room temperature, the resulting sample was treated twice with a hot aqueous solution of 2 M NaOH to remove the silica template, followed by washing with water several times and then drying at 60 °C.

The preparation of disordered mesoporous β-MnO_2_ with a pore diameter of ca. 30 nm was as follows [14]: 100 g Ludox AS-40 colloid silica (40%) was first dried at 60 °C overnight, then impregnated with 10 mL of saturated Mn(NO_3_)_2_ solution and again dried. Following this procedure it was calcined at 400 °C for 3 h. Finally the resulting material was treated twice with a 2 M hot NaOH solution, followed by washing with water several times and drying at 60 °C. This material was named β-MnO_2_-d30.

### 3.3. Materials Characterization

TEM studies were carried out using a JEOL JEM-2011 (JEOL Ltd., Tokyo, Japan), employing a LaB6 filament as the electron source and an accelerating voltage of 200 keV. TEM images were recorded by a Gatan CCD camera in a digital format. Wide-angle powder X-ray diffraction data were collected on a Stoe STADI/P powder diffractmeter operating in transmission mode and with a small angle position sensitive detector (STOE & Cie GmbH, Darmstadt, Germany). Incident radiation was generated using a Fe K_α1_ source (λ = 1.936 Å). N_2_ adsorption-desorption analysis was carried out using a Micromeritics Tristar 3020 (Micromeritics Instrument Corporation, Norcross, GA, USA). The typical sample weight used was 100–200 mg. The degassing condition was set to 180 min at 120 °C under vacuum and all adsorption-desorption measurements were carried out at liquid nitrogen temperature. Pore size distribution was analyzed by BJH model.

### 3.4. Catalytic Testing

The catalytic reaction condition could be found our previous reports [23,27,28]. Briefly, to test the performance in CO oxidation, 50 mg of catalyst was loaded in in a U-shaped quartz tube, pretreated in 8% O_2_–He at 400 °C for 1 h, and cooled down to 0 °C to allow for CO oxidation. The CO concentration was 1% (balance air), and the gas flow rate was 37 mL/min. The temperature in between 0 °C and room temperature was tuned by allowing a cup of ice water to warm naturally, and the temperature above room temperature was ramped using a furnace at a rate of 1 °C/min. The exiting stream was analyzed by GC.

To test the performance in N_2_O decomposition, 50 mg of catalyst was loaded into a U-shaped glass tube, pretreated in 20% O_2_–He at 400 °C for 1 h, and cooled down to near room temperature. The gas stream was switched to 0.5% N_2_O–He, and the flow rate was 60 mL/min. The temperature was ramped using a furnace, and kept at 100, 150, 200, 250, 300, 350, and 400 °C for 30 min at each reaction temperature. The exiting stream was analyzed by GC.

## 4. Conclusions

Mesoporous nanocrystalline β-MnO_2_ samples were successfully synthesized by nanocasting from mesoporous silica SBA-15 and KIT-6, disordered mesoporous silica, and colloidal silica. The textural properties can be tuned by varying the hydrothermal temperature of KIT-6 from 60 °C to 100 °C, or the ration of manganese nitrate to the template in the preparation. The mesoporous manganese oxide show ordered/disordered mesostructured and large surface areas. The applications in catalytic reaction of CO oxidation and decomposition of N_2_O demonstrated that mesoporous manganese oxides have superior catalytic activity than the bulk one.

## Figures and Tables

**Table 1.** Physicochemical properties of the mesoporous MnO_2_ materials ^a^.

Materials	Template	*S*_BET_ (m^2^/g)	*D* (nm)	*V* (cm^3^/g)	Pore Wall Thickness (nm, by TEM)	*T*_50_ of CO Oxidation (°C)
β-MnO_2_-60	KIT-60	83	3.6/12.8(0.80) ^a^	0.37	5.0	119
β-MnO_2_-80	KIT-80	86	3.3/12.7(0.75) ^a^	0.33	6.5	110
β-MnO_2_-100	KIT-100	84	3.4/12.8(0.43) ^a^	0.27	7.5	134
β-MnO_2_-100B	KIT-100	87	3.3/12.8(0.91) ^a^	0.30	7.5	125
β-MnO_2_-1D-100	SBA-15	68	3.4	0.26	8.8	128
β-MnO_2_-d4	Disordered mesoporous silica	135	3.4	0.44	8–10	109
β-MnO_2_-d30	AS-40	30	28	0.22	20–30	102
Bulk β-MnO_2_	Aldrich	0.5	–	–	–	400

a *S*_BET_, surface area calculated by the BET method; *D* pore diameter calculated by the BJH method (ratios of large (12.8 nm) to small (3.4 nm) pore volumes are given in parentheses); *V* total pore volume at *P*/*P*_0_ = 0.99.
